# Intratumoral CD8+ tumor-infiltrating lymphocytes as prognostic predictors in radio-chemoradiotherapy-treated nasopharyngeal carcinoma

**DOI:** 10.3389/fonc.2025.1551980

**Published:** 2025-05-15

**Authors:** Xinjing Li, Xiaoming Qiu, Cuihong Lin, Yuanying Liu, Yongbin Wang, Langlang Tang, Yuanhe Tong, Linbo Tang

**Affiliations:** ^1^ Department of Pathology, Longyan First Affiliated Hospital of Fujian Medical University, Longyan, China; ^2^ Department of Radiation Oncology, Longyan First Affiliated Hospital of Fujian Medical University, Longyan, China; ^3^ Department of Radiology, Longyan First Affiliated Hospital of Fujian Medical University, Longyan, China

**Keywords:** nasopharyngeal carcinoma, immunohistochemistry, tumor-infiltrating lymphocyte, CD4, CD8, prognosis

## Abstract

**Background:**

The prognostic value of tumor-infiltrating lymphocytes (TILs) in nasopharyngeal carcinoma (NPC) has been established. However, the prognostic significance of CD4+ and CD8+ TIL subtypes in NPC remains unclear.

**Methods:**

We collected 214 tissue samples diagnosed with NPC for immunohistochemical staining. The density of CD4+ and CD8+ TILs was evaluated in intratumoral (within tumor cell nests) and stromal (the surrounding stroma of tumor cell nests) areas. Correlations between TIL density and progression-free survival (PFS) and overall survival (OS) were analyzed.

**Results:**

High levels of intratumoral CD8+ TILs were significantly associated with reduced risk of disease progression (HR 0.382; 95% CI, 0.178-0.819, P = 0.013) and death (HR 0.265; 95% CI, 0.104-0.675, P = 0.005). Although high stromal CD8+ TIL levels were linked to higher PFS and OS, these differences did not reach statistical significance (P = 0.114 and P = 0.079, respectively). CD4+ TILs showed no significant correlation with PFS or OS. In multivariate analysis, intratumoral CD8+ TILs remained an independent prognostic factor for PFS and OS. Subgroup analysis revealed that in patients with locally advanced disease, high intratumoral CD8+ TILs were significantly associated with improved PFS (HR 0.329; 95% CI, 0.129-0.843, P = 0.021) and OS (HR 0.209; 95% CI, 0.064-0.681, P = 0.009). Conversely, in early-stage patients, neither CD8+ nor CD4+ TILs were significantly associated with PFS or OS.

**Conclusion:**

Our findings suggest that intratumoral CD8+ TILs serve as a reliable prognostic biomarker for NPC, with their prognostic value particularly pronounced in patients with locally advanced disease.

## 1 Introduction

Nasopharyngeal carcinoma (NPC) is a malignant tumor that originates from the nasopharyngeal epithelium. Although it is relatively uncommon worldwide, with an age-standardized incidence rate (ASIR) of 1.3 per 100,000 people, its incidence has significantly increased in certain regions. For instance, in Southeast Asia, the ASIR is 4.7 per 100,000. In the southeastern regions of China, the ASIR generally exceeds 10 per 100,000, while in North Africa, particularly in the Maghreb region, the ASIR reaches 3.5 per 100,000 (1). The occurrence of NPC is closely associated with Epstein-Barr virus (EBV), which induces the malignant transformation of nasopharyngeal epithelial cells through mechanisms such as viral genes, epigenetic changes, and immune evasion (2). Radiotherapy remains the primary curative treatment for NPC, and advances in radiotherapy techniques and the integration of combined anti-tumor therapies have significantly improved the cure rates. However, approximately 20% of patients still experience recurrence despite these advancements (3, 4). To reduce the probability of disease recurrence, multidisciplinary collaboration in formulating treatment strategies is particularly crucial. For patients with locally advanced disease, combining radiotherapy with chemotherapy and targeted therapy can enhance therapeutic efficacy (2). Furthermore, recent studies indicate that incorporating immunotherapy may further improve progression-free survival rates in locally advanced NPC patients (5). With rapid advancements in molecular biology, developing personalized treatment strategies for cancer patients has become increasingly feasible. Nevertheless, the traditional TNM staging system has proven inadequate in guiding clinical treatment and predicting patient prognosis (6), highlighting the urgent need for novel prognostic biomarkers.

Tumor-infiltrating lymphocytes (TILs) are a heterogeneous group of immune cells present in the tumor microenvironment, including subsets such as T cells, B cells, and NK cells. Among these, T cells play a pivotal role in regulating immune functions and mediating the destruction of tumor cells. As a result, therapeutic strategies targeting TILs have offered new hope for anti-tumor treatments (7, 8). Furthermore, numerous studies have highlighted the prognostic significance of TILs in various cancers. A meta-analysis on NPC demonstrated that higher levels of TILs were significantly associated with improved overall survival and disease-free survival (9), and similar findings have been observed in other malignancies (10–13). However, given the considerable diversity within TIL subsets, understanding their specific roles can provide deeper insights into tumor prognosis and inform more effective clinical management.

The immune characteristics of NPC are typically described as a highly immunoinflammatory environment, which is closely associated with lymphocyte infiltration (14). Single-cell analysis has shown that the proportion of T cells in the NPC tumor microenvironment is higher than in other cancers, such as non-small cell lung cancer, colorectal cancer, and pancreatic ductal adenocarcinoma, with the majority of T cells being CD4+ and CD8+ subsets (15). Previous studies have highlighted the prognostic significance of TILs in NPC (16, 17), yet the specific role of CD4+ and CD8+ TIL subtypes as prognostic biomarkers in NPC remains under investigation. Given the differences in immune cell distribution within the tumor microenvironment, this study analyzes the density of CD4+ and CD8+ TILs in intratumoral and stromal compartments and correlates them with patient survival outcomes. The goal is to clarify the prognostic value of CD4+ and CD8+ TILs in NPC.

## 2 Materials and methods

### 2.1 Patient selection

This study included patients diagnosed with NPC and treated at our hospital between January 1, 2016, and December 31, 2021. The inclusion criteria were as follows: (1) histologically confirmed diagnosis of NPC; (2) no distant metastasis at the time of diagnosis; and (3) completion of the entire treatment regimen as planned. Exclusion criteria included: (1) lack of available histological specimens; (2) history of previous malignancies; and (3) presence of severe underlying conditions. Patient information, including age, sex, and histological type, was extracted from the electronic medical record system. Patients were re-staged based on the American Joint Committee on Cancer (AJCC) 8th edition staging criteria. All patients underwent radical intensity-modulated radiotherapy, with those in clinical stages II or higher also receiving neoadjuvant or concurrent chemotherapy. The Ethics Committee of Longyan First Affiliated Hospital of Fujian Medical University approved this study.

### 2.2 Immunohistochemistry

All NPC cases were processed as formalin-fixed, paraffin-embedded (FFPE) specimens, then sectioned at a thickness of 4.0 μm for IHC examination. IHC staining was performed using a Roche Autostainer (Ventana Medical Systems, Inc.) and the OptiView DAB IHC Detection Kit (Ventana Medical Systems, Inc.) as the detection system. Prediluted primary antibodies, CD4 (SP35) and CD8 (SP16), both from Maixin BIO (China), were used for staining. The main steps of IHC are as follows: After the sections were baked at 65°C for 1 hour, they were deparaffinized with EZ Prep deparaffinization solution. Heat-induced antigen retrieval was performed using CC1 solution at 100°C for 52 minutes. Blocking was done with 3% H2O2 at 37°C for 4 minutes. Primary antibody (100 µL) was added and incubated at 37°C for 36 minutes. The secondary antibody (HRP Multimer) was incubated at 37°C for 8 minutes. Color development was performed using 0.2% DAB and 0.04% H2O2 at 37°C for 8 minutes, followed by incubation with hematoxylin and bluing solution.

### 2.3 Quantification of tumor-infiltrating lymphocytes

TIL infiltration was assessed by semi-quantitative estimation of the densities of CD4+ and CD8+ cells, with scoring conducted for both intratumoral areas (cells within tumor cell nests) and stroma (the surrounding stroma of tumor cell nests). Areas of necrosis and submucosal intrinsic lymphoid tissue were excluded from the analysis. For stromal area TIL infiltration assessment, this study referred to methods reported in previous literature (18, 19), where the denominator used to determine the percentage of stromal TILs is the area of stromal tissue. The specific scoring criteria are as follows: 1, no or sporadic cells (≤10% stromal TILs); 2, a small number of cells (>10% stromal TILs and ≤30% stromal TILs); 3, abundant occurrence of cells (>30% stromal TILs and ≤50% stromal TILs); 4, highly abundant occurrence of cells (>50% stromal TILs). For the tumor nest area, scoring was based on the proportion of positive cells relative to all nucleated cells within each selected field. The criteria were: 1, no or sporadic cells (positive cell count <5%); 2, a small number of cells (positive cell count >5% and ≤15%); 3, abundant occurrence of cells (positive cell count >15% and ≤30%); 4, highly abundant occurrence of cells (positive cell count >30%). To ensure consistency, three random fields with the highest TIL density were selected for evaluation, with each section independently scored by two pathologists blinded to clinicopathological data. In cases of scoring discrepancies, the pathologists reviewed the sections together and reached a consensus. Based on the TIL scores, the cohort was categorized into two groups: low (scores 1–2) and high (scores 3–4).

### 2.4 Statistical analyses

Patient data on disease progression and survival outcomes were obtained from the electronic medical record system and telephone follow-up. The study endpoints were progression-free survival (PFS), defined as the time from diagnosis to disease progression or death, and overall survival (OS), defined as the time from diagnosis to death from any cause. The Kaplan-Meier method was used to estimate PFS and OS, and the log-rank test was employed to assess differences between groups. Univariate and multivariate analyses were conducted using Cox regression. A two-sided P value of less than 0.05 was considered statistically significant. All statistical analyses were performed using SPSS (version 25.0) and R (version 4.4.1).

## 3 Results

### 3.1 Patient characteristics

The clinicopathological characteristics of the 214 NPC patients included in this study are summarized in **Table 1**. The median age of the patients was 51 years (range: 21–84 years), with the majority being male (73.8%). The most common pathological type was non-keratinizing carcinoma (WHO type II), which accounted for 96.3% of all cases. Among the patients, 74.8% were classified as having locally advanced disease (clinical stage III-IV). 92.5% of patients received platinum-based chemotherapy, while the remaining 7.5% received only radical intensity-modulated radiotherapy.

**Table 1:** Clinicopathological characteristics of 214 patients with nasopharyngeal carcinoma.

Variables	No. (%)
Age, median (IQR)	51(43.5-62.5)
Sex	
Female	56(26.2%)
Male	158(73.8%)
Pathology	
Non-keratinizing (WHO II)	206 (96.3%)
Others	8(3.7%)
T-category	
T1-2	102 (47.7%)
T3-4	112 (52.3%)
N-category	
N0-1	96(44.9%)
N2-3	118 (55.1%)
Clinical stage	
I-II	54(25.2%)
III-IV	160(74.8%)
Radiation dose	
≤68Gy	27 (12.6%)
>68Gy	187 (87.4%)
Chemotherapy	
No	16 (7.5%)
Yes	198 (92.5%)
CD8+TILs (intratumoral)	
high	72(33.6%)
low	142(66.4%)
CD8+TILs (stromal)	
high	57(26.6%)
low	157(73.4%)
CD4+TILs (intratumoral)	
high	80(37.4%)
low	134(62.6%)
CD4+TILs (stromal)	
high	64(29.9%)
low	150(70.1%)

IQR, Interquartile range; TILs, tumor-infiltrating lymphocytes.

### 3.2 CD4+ and CD8+ TIL expression

**Figure 1:**
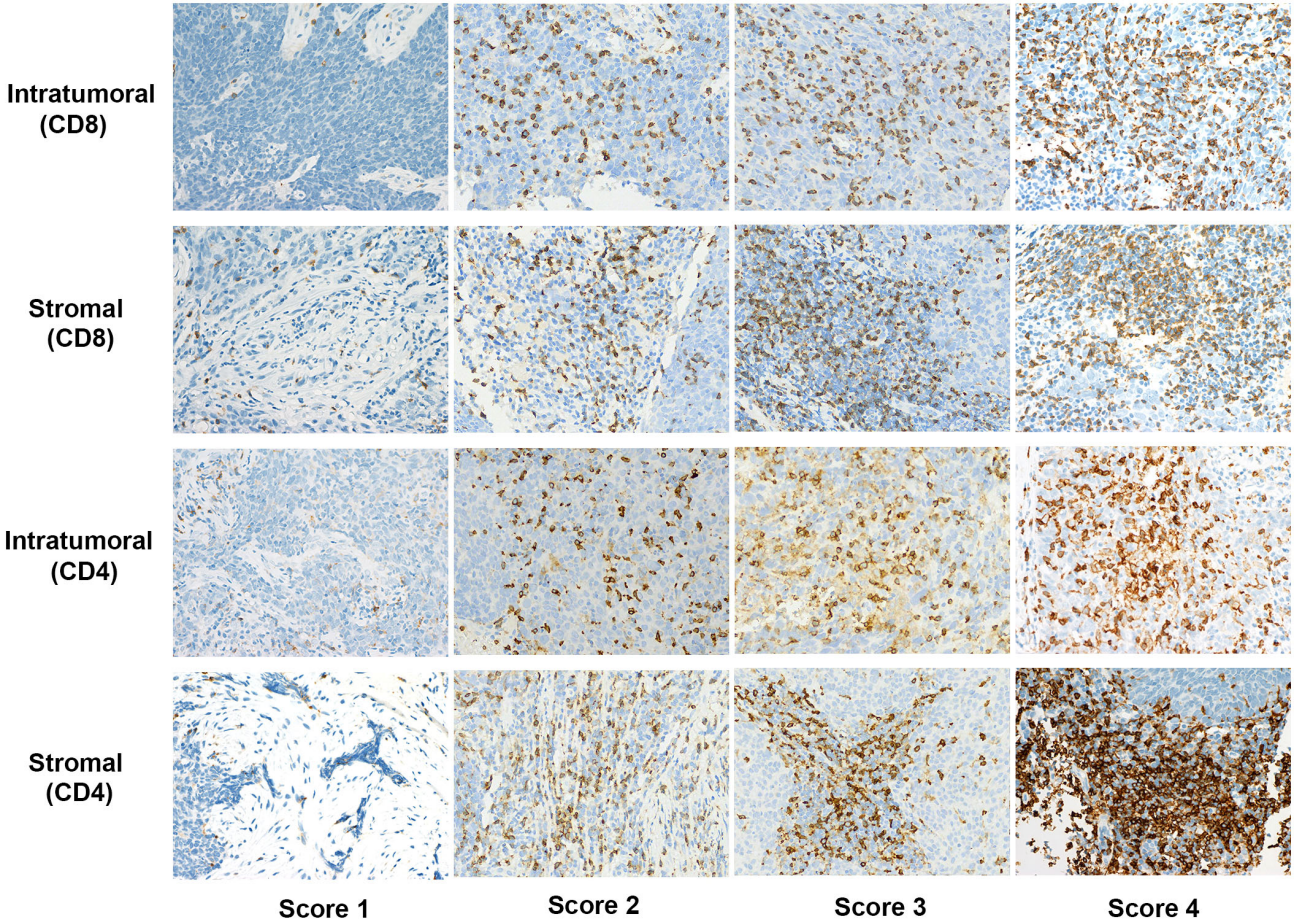
Representative immunohistochemical staining scores of CD4 and CD8 expression in intratumoral and stromal compartments at 400x magnification.

Immunohistochemistry results revealed 100% positivity for both CD8+ and CD4+ TILs. Further analysis showed that in the intratumoral compartment, 33.6% of patients had high levels of CD8+ TILs, while 66.4% had low levels. In the stromal compartment, 26.6% exhibited high levels of CD8+ TILs, while 73.4% had low levels. Regarding CD4+ TILs, 37.4% of patients showed high intratumoral density, while 29.9% demonstrated high density in the stroma. Representative immunohistochemical staining images of CD8+ and CD4+ TILs in both compartments are shown in **Figure 1**.

### 3.3 Survival outcomes and prognostic analysis

As of the last follow-up on August 30, 2024, the median follow-up was 57.5 months. A total of 17 patients (7.9%) experienced recurrence in the nasopharynx or cervical lymph nodes, 29 patients (13.6%) developed distant metastasis, and 41 patients (19.2%) died. The 5-year PFS for the entire cohort was 77.0%, and the 5-year OS was 80.9%.

**Figure 2:**
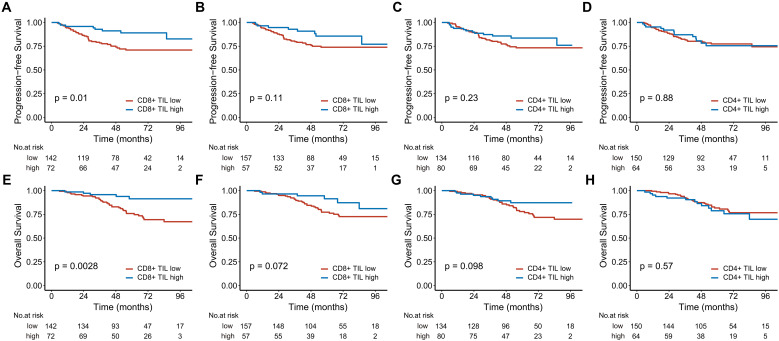
Kaplan-Meier estimates of progression-free survival **(A-D)** and overall survival **(E-H)** based on CD8+ and CD4+ TIL levels in the entire cohort are shown. Panels **(A, C, E)**, and **(G)** represent the intratumoral compartment, while panels **(B, D, F)**, and **(H)** represent the stromal compartment. TIL, tumor-infiltrating lymphocyte.

The 5-year PFS of patients with high intratumoral CD8+ TILs was 82.7%, significantly higher than the 71% observed in those with low intratumoral CD8+ TILs (**Figure 2A**). High intratumoral CD8+ TILs were associated with a significantly reduced risk of disease progression (HR: 0.382; 95% CI: 0.178–0.819, P = 0.013). In contrast, while patients with high stromal CD8+ TILs showed a better 5-year PFS compared to those with low stromal CD8+ TILs (85.6% vs. 73.9%), this difference did not reach statistical significance (P = 0.11, **Figure 2B**). The 5-year PFS for patients with high and low intratumoral CD4+ TILs was 83.6% and 74.4%, respectively (P = 0.23, **Figure 2C**). Similarly, the 5-year PFS for patients with high and low stromal CD4+ TILs was 75.5% and 79.3%, respectively (P = 0.88, **Figure 2D**). After adjusting for age, T stage, and N stage in the multivariate analysis, only intratumoral CD8+ TILs were identified as an independent prognostic factor for PFS (HR: 0.431; 95% CI: 0.189–0.985, P = 0.046) (**Table 2**).

**Table 2:** Univariate and multivariable Cox regression analysis for progression-free survival.

Factor		Univariate analysis		Multivariate analysis	
		HR (95%CI)	*P*	HR (95%CI)	*P*
Age (year)	≤51	1		1	
	>51	1.706(0.947-3.071)	0.075	1.535(0.834-2.825)	0.168
Sex	Male	1			
	Female	1.216(0.649-2.279)	0.542		
Pathology	WHO II	1			
	Others	0.047(0.000-36.377)	0.367		
T-category	T1-2	1		1	
	T3-4	2.182(1.176-4.050)	0.013	1.996(1.058-3.768)	0.033
N-category	N0-1	1		1	
	N2-3	1.969(1.050-3.691)	0.035	1.746(0.912-3.344)	0.093
Radiation dose	≤68Gy	1			
	>68Gy	1.413(0.556-3.592)	0.467		
Chemotherapy	No	1			
	Yes	3.290(0.453-23.869)	0.239		
CD8+TILs^a^	low	1		1	
	high	0.382(0.178-0.819)	0.013	0.431(0.189-0.985)	0.046
CD8+TILs^b^	low	1		1	
	high	0.541(0.252-1.159)	0.114	0.759(0.340-1.693)	0.500
CD4+TILs^a^	low	1		1	
	high	0.675(0.355-1.282)	0.230	1.064(0.518-2.186)	0.866
CD4+TILs^b^	low	1		1	
	high	0.951(0.500-1.808)	0.878	1.296(0.661-2.541)	0.450

TILs, tumor-infiltrating lymphocytes; ^a^ intratumoral; ^b^ stromal.

Patients with high intratumoral CD8+ TILs had a 5-year OS of 91.4%, while those with low intratumoral CD8+ TILs had a 5-year OS of 81% (**Figure 2E**). High intratumoral CD8+ TILs significantly reduced the risk of death (HR, 0.265; 95% CI, 0.104-0.675, P = 0.005). The 5-year OS for patients with high stromal CD8+ TILs was also higher than that for patients with low stromal CD8+ TILs, although the difference approached statistical significance (5-year OS: 91.4% vs 82.8%, P = 0.072, **Figure 2F**). The 5-year OS for patients with high and low intratumoral CD4+ TILs was 87.2% and 79.0%, respectively (P = 0.098, **Figure 2G**). For stromal CD4+ TILs, the 5-year OS was 78.8% in patients with high expression and 81.7% in those with low expression (P = 0.57, **Figure 2H**). After adjusting for age, chemotherapy status, T stage, and N stage in multivariate analysis, both intratumoral CD8+ TILs (HR 0.312; 95% CI, 0.114-0.858, P = 0.024) and stromal CD4+ TILs (HR 2.164; 95% CI, 1.074-4.360, P = 0.031) were identified as independent prognostic factors for OS (**Table 3**).

**Table 3:** Univariate and multivariable Cox regression analysis for overall survival.

Factor		Univariate analysis		Multivariate analysis	
		HR (95%CI)	*P*	HR (95%CI)	*P*
Age (year)	≤51	1		1	
	>51	4.727(2.251-9.925)	0.000	3.911(1.780-8.591)	0.001
Sex	Male	1			
	Female	0.524(0.232-1.182)	0.120		
Pathology	WHO II	1			
	Others	0.686(0.094-4.993)	0.710		
T-category	T1-2	1		1	
	T3-4	2.368(1.224-4.581)	0.010	2.274(1.135-4.556)	0.020
N-category	N0-1	1		1	
	N2-3	2.329(1.167-4.648)	0.017	3.409(1.567-7.416)	0.002
Radiation dose	≤68Gy	1			
	>68Gy	1.259(0.524-3.026)	0.607		
Chemotherapy	No	1		1	
	Yes	0.265(0.122-0.574)	0.001	0.166(0.068-0.405)	0.000
CD8+TILs^a^	low	1			
	high	0.265(0.104-0.675)	0.005	0.312(0.114-0.858)	0.024
CD8+TILs^b^	low	1		1	
	high	0.461(0.194-1.095)	0.079	0.728(0.293-1.814)	0.496
CD4+TILs^a^	low	1		1	
	high	0.540(0.257-1.132)	0.103	1.338(0.571-3.135)	0.503
CD4+TILs^b^	low	1		1	
	high	1.208(0.626-2.334)	0.573	2.164(1.074-4.360)	0.031

TILs, tumor-infiltrating lymphocytes; ^a^intratumoral; ^b^stromal.

### 3.4 Subgroup analysis

**Figure 3:**
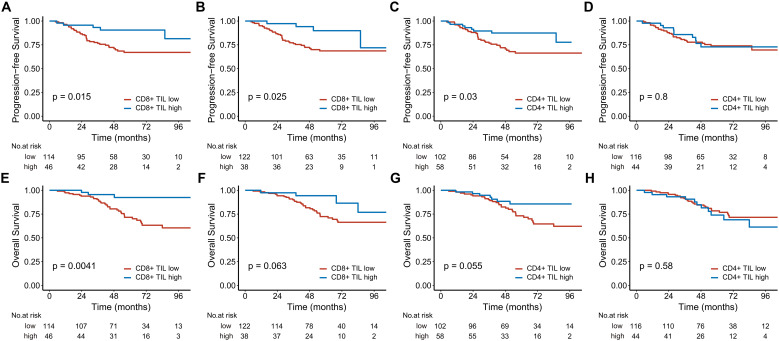
Kaplan-Meier estimates of progression-free survival **(A-D)** and overall survival **(E-H)** based on CD8+ and CD4+ TIL levels in patients with locally advanced disease. **(A, C, E)**, and **(G)** represent the intratumoral compartment, while **(B, D, F)**, and **(H)** represent the stromal compartment. TIL, tumor-infiltrating lymphocyte.

**Figure 4:**
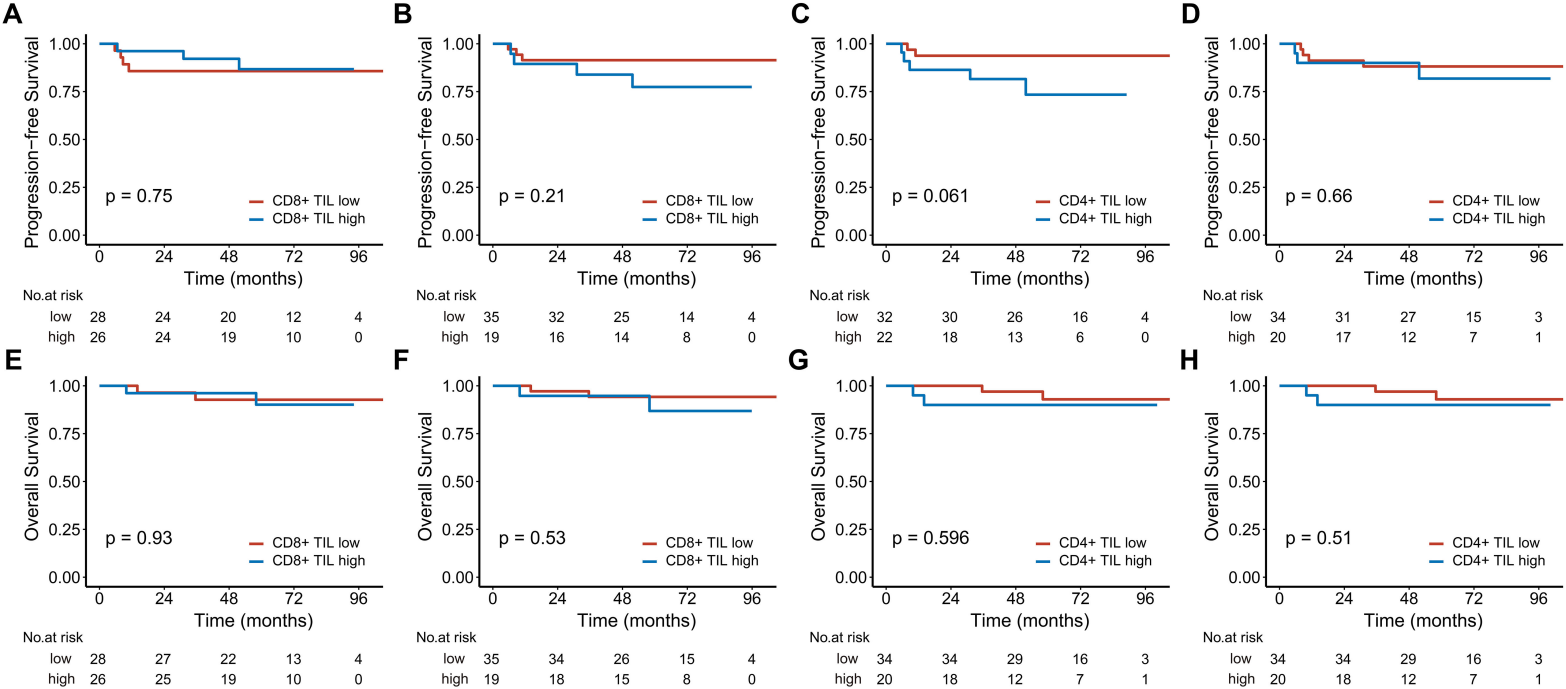
Kaplan-Meier estimates of progression-free survival **(A-D)** and overall survival **(E-H)** based on CD8+ and CD4+ TIL levels in early-stage patients. **(A, C, E)**, and **(G)** represent the intratumoral compartment, while **(B, D, F)**, and **(H)** represent the stromal compartment. TIL, tumor-infiltrating lymphocyte.

In the analysis of 160 stage III-IV patients, high intratumoral CD8+ TILs significantly reduced the risks of disease progression (HR 0.329; 95% CI, 0.129-0.843, P = 0.021) and death (HR 0.209; 95% CI, 0.064-0.681, P = 0.009). High intratumoral CD4+ TILs were associated with better 5-year PFS (**Figure 3**). In contrast, for the 54 stage I-II patients, neither CD8+ nor CD4+ TILs demonstrated significant associations with PFS or OS (**Figure 4**).

## 4 Discussion

TILs have long been considered prognostic markers for malignant tumors; however, the findings in the literature have not always been consistent (20–22). The reasons for this inconsistency are multifactorial. On the one hand, different types of tumors exhibit distinct immune characteristics, leading to variations in the types and abundance of immune cells within the tumor microenvironment (23). Additionally, the distribution of immune cells both within the tumor nests and its surrounding stromal tissue may further influence their prognostic significance. In this study, we detected CD4+ and CD8+ TILs infiltration in all patients, with density varying between the intratumoral and stromal compartments. Patients with high intratumoral CD8+ TILs showed better PFS and OS, whereas no significant survival differences were found based on stromal CD8+ TIL levels. Neither intratumoral nor stromal CD4+ TILs demonstrated a significant association with prognosis in univariate analysis. Multivariate analysis identified intratumoral CD8+ TILs as the only independent prognostic factor for PFS and OS.

High-density TILs are generally associated with a favorable prognosis in malignant tumors (24–26). A meta-analysis of 3,708 NPC patients demonstrated that high TIL levels significantly reduce the risk of death (9), and similar findings were confirmed in another large cohort study (16). However, given the presence of functionally diverse TIL subpopulations, which play crucial roles in both immune activation and suppression, it is more meaningful to elucidate the prognostic value of specific subgroups. CD8+ TILs, in particular, are the primary effector cells responsible for cytotoxicity, capable of killing tumor cells through the secretion of perforin, granzyme B, and IFN-γ (27, 28).

Previous studies have reported that high CD8+ TILs are associated with a favorable prognosis in various tumors (29, 30), which is unsurprising, as the abundant presence of CD8+ T cells can enhance the host’s anti-tumor immunity and aid in tumor elimination. Shi et al. (31) investigated the relationship between CD8+ TILs and prognosis in NPC. Their findings revealed that high CD8+ TILs in the tumor parenchyma were significantly associated with improved overall and disease-free survival, a correlation confirmed in both the training and validation cohorts. However, no significant association was found between stromal CD8+ TILs and prognosis. These results are consistent with our findings, but our study has a larger sample size. In our study, high intratumoral CD8+ TILs were significantly associated with a reduced risk of disease progression and death. Although stromal CD8+ TILs also showed a similar trend, the difference was insignificant. This may be because CD8+ T cells migrating into the tumor nests more effectively exert cytotoxic effects. For example, a study analyzing the impact of the average distance between CD8+ T cells and tumor cells in the tumor microenvironment showed that the subgroup with the closest distance had significantly better overall survival and disease-free survival (32). Interestingly, a retrospective analysis by Wang et al. (16) suggested that stromal TILs were superior to intratumoral TILs as prognostic factors in NPC. However, since this study counted TILs on hematoxylin and eosin (H&E)-stained slides, the impact of TIL subpopulations on prognosis was not analyzed.

CD4+ T cells are another important subpopulation of TILs. Traditionally, CD4+ TILs were believed to function in immune regulation during tumor resistance primarily. However, recent studies have shown that CD4+ T cells can also act directly as effector cells, eliminating tumor cells through mechanisms similar to the cytotoxic actions of CD8+ T cells (33). Although the response intensity is lower than that of CD8+ T cells, a sufficient number of CD4+ TILs can still exert significant tumor-killing effects (34). Several studies have reported that CD4+ TILs are associated with prognosis in various tumors (22, 35), but research on the prognostic significance of CD4+ TILs in NPC remains limited. Liu et al. (9) summarized data from two small-sample studies and found that high CD4+ TILs were associated with better OS, although this result was not confirmed in subsequent studies (31, 36). In our study, neither intratumoral nor stromal CD4+ TILs significantly associated with PFS or OS in the entire cohort. However, in the subgroup analysis of locally advanced patients, high intratumoral CD4+ TILs were significantly associated with improved PFS. Given the diversity of CD4+ TIL subpopulations and their functions, further investigation into their prognostic value is needed.

Our subgroup analysis found that for early-stage patients, neither CD8+ nor CD4+ TILs showed a significant association with PFS or OS. This may be because early-stage NPC generally responds well to treatment. A long-term follow-up study has indicated that the 10-year survival rate for early-stage NPC can reach approximately 90% (37), making it more challenging to stratify these patients prognostically using biomarkers. In contrast, for locally advanced patients, high levels of intratumoral CD8+ TILs were significantly associated with improved PFS and OS. Furthermore, their ability to reduce the risk of disease progression or death (hazard ratio, HR) was more pronounced than the entire cohort analysis. Therefore, we infer that the prognostic value of intratumoral CD8+ TILs is more effectively realized in locally advanced NPC patients, which could provide valuable insights for the clinical management of NPC.

It is important to note that this study has several limitations. First, as a retrospective study, information bias is inevitable. Additionally, all patients were from the same institution, which limits the generalizability of the results to different regional populations. Furthermore, chemotherapy is an independent prognostic factor affecting OS, and the heterogeneity of chemotherapy regimens used by patients may have impacted the results. However, given that most patients received platinum-based chemotherapy, this potential bias is likely minimized. Finally, it should be noted that all patients in this study cohort received only chemoradiotherapy, without the involvement of immunotherapy. Given that immune checkpoint inhibitors (ICIs) and other immunotherapeutic agents are known to remodel the tumor microenvironment (TME), this limitation precludes our ability to assess how such interventions might influence the spatial distribution and prognostic relevance of TILs within the TME.

In conclusion, our study reinforces the findings of most existing reports regarding the prognostic significance of TILs. While TNM staging remains the primary determinant of prognosis in NPC, intratumoral CD8+ TILs may serve as a complementary biomarker to optimize risk stratification, particularly in patients with locally advanced disease. This risk stratification is critically important for developing personalized treatment strategies, as it helps identify patients who may benefit from intensified therapies. Given the close relationship between the occurrence of NPC and EBV, which may affect the immune microenvironment of NPC, future studies should integrate EBV status analysis to elucidate its potential regulatory role in TIL functionality and prognosis.

## Data Availability

The raw data supporting the conclusions of this article will be made available by the authors, without undue reservation.
